# Synergism of Cyclin-Dependent Kinase Inhibitors with Camptothecin Derivatives in Small Cell Lung Cancer Cell Lines

**DOI:** 10.3390/molecules19022077

**Published:** 2014-02-17

**Authors:** Gerhard Hamilton, Lukas Klameth, Barbara Rath, Theresia Thalhammer

**Affiliations:** 1 Ludwig Boltzmann Cluster of Translational Oncology, Vienna A-1090, Austria; E-Mails: lukas.klameth@toc.lbg.ac.at (L.K.); barabara.rath@meduniwien.ac.at (B.R.); 2 Department of Surgery, Medical University of Vienna, Vienna A-1090, Austria; 3 Department of Pathophysiology and Allergy Research, Center for Pathophysiology, Infectiology and Immunology, Medical University of Vienna, Vienna A-1090, Austria; E-Mail: theresia.thalhammer@meduniwien.ac.at

**Keywords:** small cell lung cancer, camptothecin, topotecan, rubitecan, cyclin-dependent kinase inhibitor, PD0332991, roscovitine, olomoucine, chemoresistance, MTT assay, cell cycle, topoisomerase I

## Abstract

Advanced small cell lung cancer (SCLC) has a dismal prognosis. Modulation of the camptothecin topotecan, approved for second-line therapy, may improve response. Our recent finding of synergistic enhancement of the cytotoxic activity of camptothecin (CPT) by cyclin-dependent kinase 4 inhibitors is extended here to a panel of camptothecin analogs comprising 10-hydroxy-CPT (HOCPT), topotecan (TPT; 9-[(dimethylamino)-methyl]-10-hydroxy-CPT), 9-amino-CPT (9AC), 9-nitrocamptothecin (rubitecan), SN38 (7-ethyl-10-hydroxycamptothecin) and 10-hydroxy-9-nitrocamptothecin (CPT109) in combination with PD0332991, CDK4I, roscovitine and olomoucine. SCLC cell lines employed are chemoresistant NCI-H417 and DMS153 and the chemosensitive SCLC26A line established at our institution. The CPT analogs exhibiting highest cytotoxicity towards the three SCLC lines tested were SN38 and 9AC, followed by rubitecan, HOCPT, TPT and CPT109. NCI-H417 and DMS153 revealed an approximately 25-fold and 7-fold higher resistance compared to the chemosensitive SCLC26A cell line. Whereas the CDK4/6 inhibitor PD0332991 proved less effective to chemosensitize SCLC cells to CPT analogs, the CDK inhibitors CDK4I, roscovitine and olomoucine gave comparable chemosensitization effects in combination with 9AC, SN38, rubitecan and to a lesser extent with TPT and CPT109, not directly related with topoisomerase mRNA expression. In conclusion, small chemical modifications of the parent CPT structure result in differing cytotoxicities and chemomodulatory effects in combination with CDKIs of the resulting analogs.

## 1. Introduction

Small cell lung cancer (SCLC) is a highly malignant neuroendocrine tumor of the lung accounting for approximately 13% of all lung cancer diagnoses and its treatment poses a challenge because of its rapid growth, early dissemination and development of drug resistance during the course of the disease [1,2,3]. Without treatment, SCLC has a median survival from diagnosis as low as 2 to 4 months. The standard combination of etoposide and cisplatin/carboplatin chemotherapy with concurrent chest radiation therapy achieves median survivals of 18 to 24 months, but despite high initial response rates the majority of patients relapse early and exhibit chemoresistance. 

Topotecan (TPT), a water soluble semisynthetic derivative of camptothecin (CPT), has demonstrated antineoplastic activity and is currently the only agent approved for second-line therapy in SCLC [4]. The drug targets the nuclear enzyme topoisomerase I (TOP1) which relaxes supercoiled DNA and the mechanism of CPT poisoning of TOP1 rests on inhibition of the religation function of the enzyme, resulting in the stabilization of the TOP1-cleavable complexes leading to fork stalling and the formation of DNA double-strand breaks in proliferating cells upon DNA readout [4,5]. As with most chemotherapeutics, intrinsic and acquired drug resistance represents an obstacle that limits the success of therapy with CPT analogs. 

**Figure 1:**
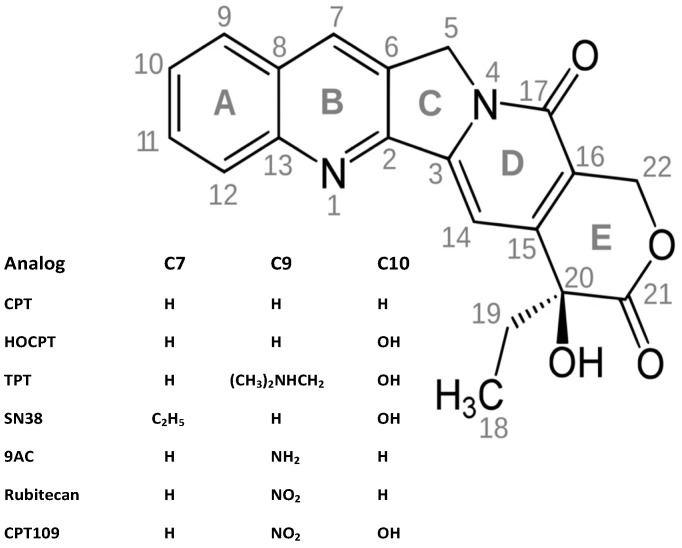
Structure of camptothecins used in the present study.

Wall and Wani isolated 20-(*S*)-camptothecin (CPT) in 1966 from the bark of *Camptotheca acuminata,* but clinical use was impeded by its poor stability and solubility [6]. The CPTs are cytotoxic quinoline alkaloids characterized by a planar pentacyclic ring system (Figure 1) Modifications of the A-D rings of CPT retain activity, while the E-ring lactone is necessary for activity as it represents the binding site for TOP I. Dependent on pH, CPTs are in equilibrium between the closed, active lactone and the open, inactive carboxylate form. To overcome the solubility and stability issues of CPTs, various derivatives have been developed, of which only two, namely irinotecan (CPT11) and topotecan, are approved for clinical use in colon cancer and different tumor entities, respectively [7,8]. Like CPT, 10-hydroxycamptothecin (HOCPT) is naturally occurring in the respective plant sources [9]. Increased solubility and applicability of TPT is due to a tertiary amine at the 9-position. The prodrug irinotecan, which has an ethyl substituent at position 7 and a dipiperidyl carbamate at position 10, is metabolized to SN38, a 7-ethyl-10-hydroxy derivative, that exhibits up to 1,000-fold increased cytotoxicity compared to the parent drug [10]. Another derivative, 9-amino-camptothecin (AC) showed the highest activity in cell culture and antitumor activity *in vitro* and *in vivo* [11]. Since clinical investigations of 9-AC showed little promise, an intermediate in its synthesis, 9-NC, was tested for cytotoxic properties and was found to be converted to 9-AC *in vivo* [12]. Although the drug showed limited solubility, it was further tested clinically as an orally available CPT. Finally, 10-hydroxy-9-nitrocamptothecin, used here for comparison, combines the two modifications in position 9 and 10 and this compound was not developed for clinical use. This panel of seven CPTs was investigated for their anticancer activity against three SCLC cell lines with different degrees of chemoresistance in the present study. We have recently demonstrated that combinations of TPT with cyclin-dependent kinase (CDK) inhibitors olomoucine, roscovitine and CDK4I exhibit synergistic cytotoxic activity against SCLC cell lines [13]. Recent developments of diverse CDk inhibitors has led to renewed interest in clinical trials for this class of agents. For example, palbociclib (formerly known as PD-0332991), a novel oral selective inhibitor of CDK4/6 that blocks tumor cell progression, has received a “breakthrough therapy designation” from the FDA for the treatment of patients with breast cancer, and similar drugs, like dinaciclib (SCH727965/targeting CDK1,2,5,9), alvocidib (flavopiridol/ CDK1,2,4,6) and seliciclib/CYC202 (roscovitine/CDK2,5) are at various phases of clinical testing [14]. Thus, in the present study, CDK inhibitors were tested for putative chemosensitizing effects in combination with this range of CPT analogs.

## 2. Results and Discussion

### 2.1. Cytotoxicity of CPT and Analogs against SCLC Cell Lines

**Figure 2:**
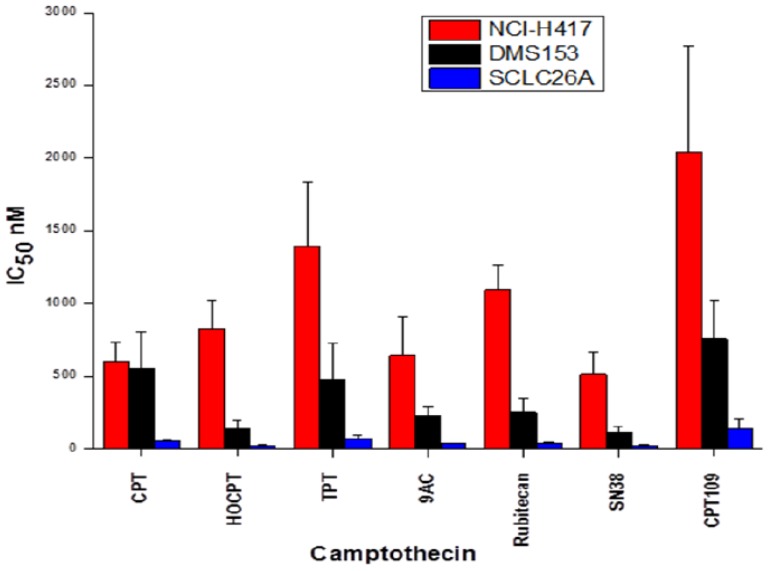
CPT, 10-hydroxy-CPT (HOCPT), topotecan (TPT), 9-aminocamptothecin (9AC), rubitecan, SN38 and CPT109 were tested for their cytotoxicity against NCI-H417, DMS153 and SCLC26A SCLC cell lines using MTT assays. IC_50_ values are presented as mean ± SEM (*n* = 3).

CPT, 10-hydroxy-CPT (HOCPT), topotecan (TPT), 9-aminocamptothecin (9AC), rubitecan, SN38 and CPT109 were tested for their cytotoxicity against NCI-H417, DMS153 and SCLC26A SCLC cell lines using MTT assays (Figure 2). Despite no prior treatment NCI-H417 is a highly chemoresistant and aggressive SCLC line, DMS153, pretreated *in vivo* with cytoxan (cyclophosphamide) and methotrexate, shows intermediate resistance and the SCLC26A cell line, established in our lab from the pleural effusion of an untreated patient, proved to be chemosensitive. In detail, NCI-H417 is 25 ± 12.4-fold and DMS153 7.2 ± 1.8-fold more resistant for all CPT analogs (mean values ± SD) in comparison to the SCLC26A cell line. Among the CPT analogs, SN38, 9AC and HOCPT exhibited the highest cytotoxicity, followed by rubitecan, topotecan and CPT109.

### 2.2. Cell Cycle Effects of CPT and Analogs on NCI-H417 and DMS153 Cell Lines

**Figure 3:**
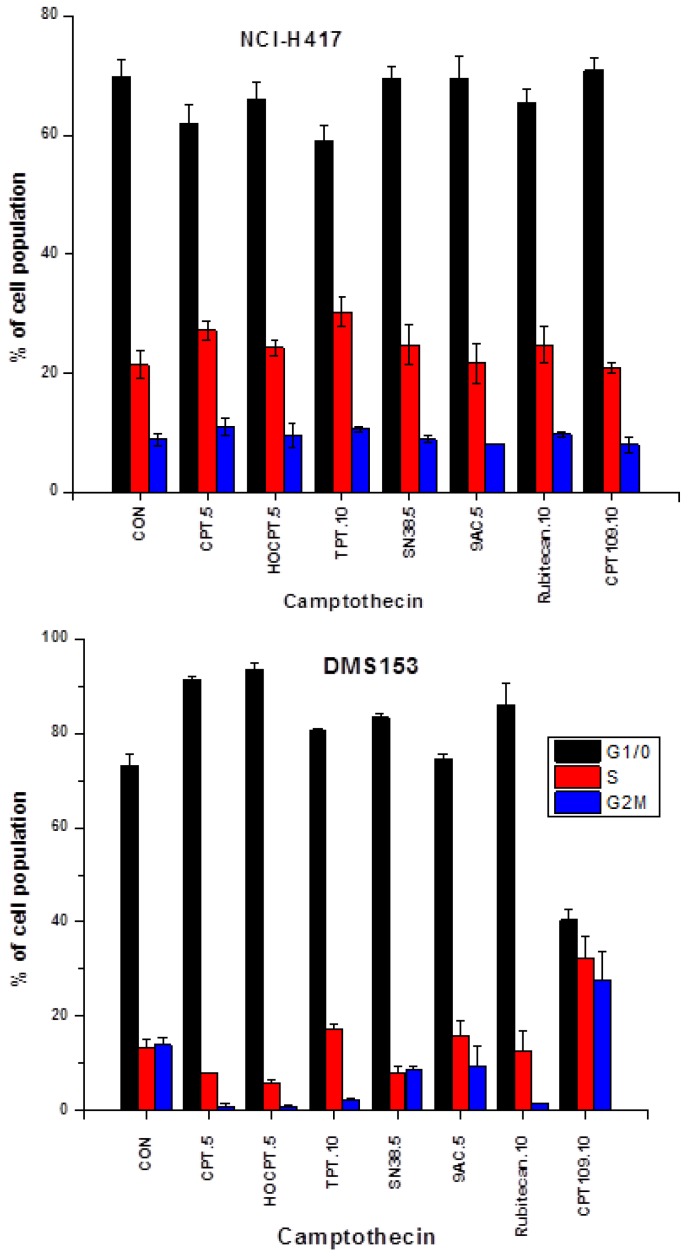
Cells were treated with the indicated concentrations of the CPT analogs (µM) in tissue culture for three days and the fixed and stained with propidium iodide for flow cytometric analysis (mean values ± SD).

Cells were treated with the indicated concentrations of the CPT analogs (µM) in tissue culture for three days and then fixed and stained with propidium iodide for flow cytometric analysis (Figure 3). In chemoresistant NCI-H417 cells CPT, HOCPT, TPT and rubitecan showed accumulation of the cells in S phase with a reduction of cells in G1/0, whereas SN38, 9AC and CPT109 did not alter cell cycle distribution significantly. In contrast, the less chemoresistant DMS153 cells showed accumulation of the cells in G1/0, except for 9AC lacking significant effects and CPT109, causing arrest of the cells in G2M phase.

### 2.3. CPT Analogs-induced Apoptotic Cell Death in DMS153 Cells

**Figure 4:**
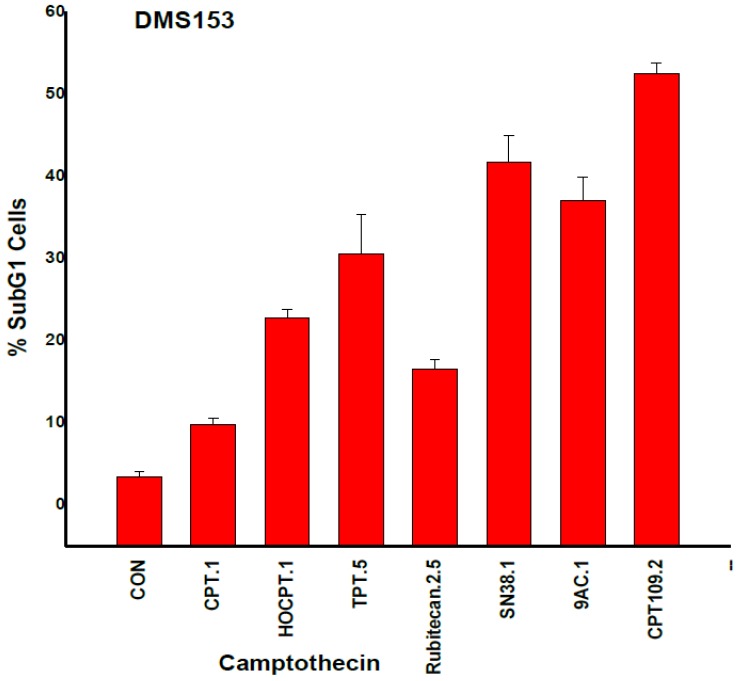
Apoptotic DMS153 cells were detected as SubG1 cells in cell cycle analyses of CPT analog-treated cultures (mean values ± SD; all values significantly different, except for SN38 and 9AC).

Apoptotic DMS153 cells were detected as subG1 cells in cell cycle analyses of CPT analog-treated cultures (Figure 4). CPT, rubitecan and HOCPT treatment resulted in a lower number of apoptotic subG1 cells compared to CPT109, SN38, 9AC and TPT. These results lack correlation with IC_50_ values as measured in MTT assays (Figure 4).

### 2.4. Effects of CDK Inhibitors on Cell Cycle Distribution of NCI-H417 and DMS153 Cells

**Figure 5:**
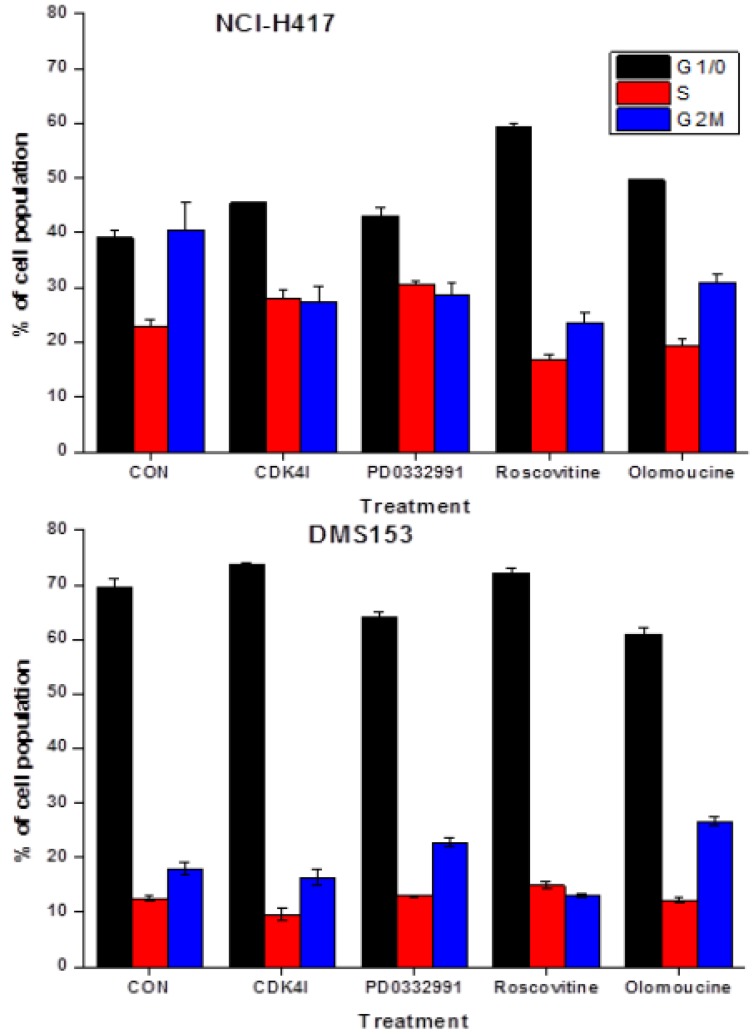
The different CDK inhibitors employed in this study were assayed for cell cycle effects in NCI-H417 and DMS153 cells. Increases in G1/0 were significant for all CDK inhibitors, except for PD0332991, and for the reduction in G2M for all inhibitors for the NCI-H417 cells. In DMS153 cells, G1/0 was significantly increased in response to CDK4I, reduced in response to PD0332991 and olomoucine, whereas G2M was increased for PD0332991 and olomoucine, but reduced for roscovitine.

The different CDK inhibitors employed in this study were assayed for cell cycle effects in NCI-H417 and DMS153 cells (Figure 5). CDK4I at 2.5 µM arrested NCI-H417 at G1/0-S phase and DMS153 at G2M-G1/0, PD0332991 at 2.5 µM arrested NCI-H417 in G1/0-S phase and DMS153 in G2M. Roscovitine at 10 µM and olomoucine at 25 µM accumulated NCI-H417 in G1/0 and DMS153 in G1/0 and G2M, respectively. Concentration of CDKIs were selected to exhibit a direct cytotoxic effect of <15% in MTT assays.

### 2.5. Chemosensitizing Effects of CDK Inhibitors on CPT Analogs in NCI-H417 and DMS153 Cells

**Figure 6:**
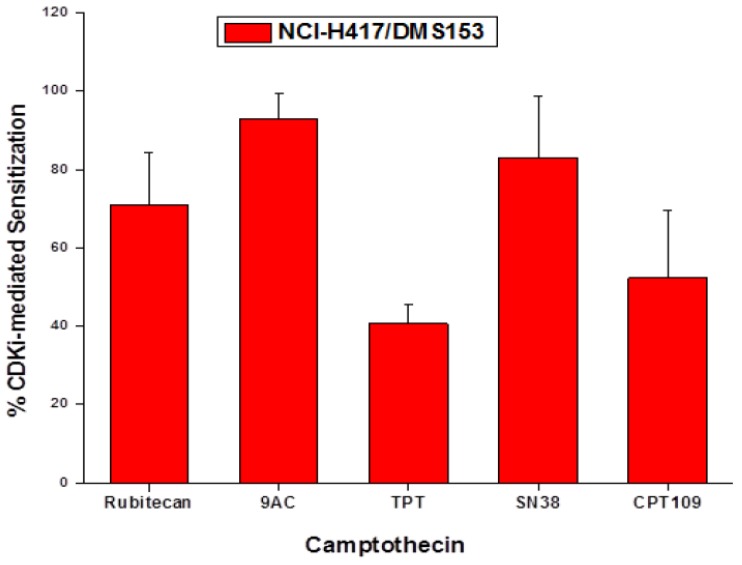
IC_50_ values for all CPT analogs in absence or presence of the respective inhibitors were calculated from dose-response curves. Since the CDK inhibitors exhibited no cytotoxic activity themselves at concentrations which showed synergistic effects with CPT cytotoxicity, the shift in IC_50_ values was measured and related to the IC_50 _values of the respective CPT analog (mean percentage of IC_50_ ± SD, *n* = 6).

The SCLC cell lines were treated with the clinically applicable CPT analogs and CPT109 alone and in combination with the four CDK inhibitors in 10 twofold dilution steps. Initial concentrations of CDKIs showed cytotoxic effects of <15% in MTT assays. IC_50_ values for all CPT analogs in absence or presence of the respective inhibitors were calculated from dose-response curves. Since the CDK inhibitors exhibited no cytotoxic activity themselves at concentrations which showed synergistic effects with CPT cytotoxicity, the shift in IC_50_ values was measured and related to the IC_50 _values of the respective CPT analog. The CDK inhibitors CDK4I, roscovitine and olomoucine revealed similar chemosensitizing effects in NCI-H417 and DMS153 cell lines and, therefore, the mean values of the reduction in IC_50_ values for the different CPT analogs and the three CDK inhibitors is presented in Figure 6. Highest chemosensitzing activity of the three CDK inhibitors was detected in combination with 9AC and SN38, followed by rubitecan, CPT109 and TPT. PD0332991 showed low chemosensitizing activity in all three cell lines used (data not shown). In chemosensitive SLCL26A cells, the CDK inhibitors had low sensitizing effects, except in combination with TPT (data not shown).

### 2.6. Effects of the CDK Inhibitors on Topoisomerase I Expression of DMS153 Cells

**Figure 7:**
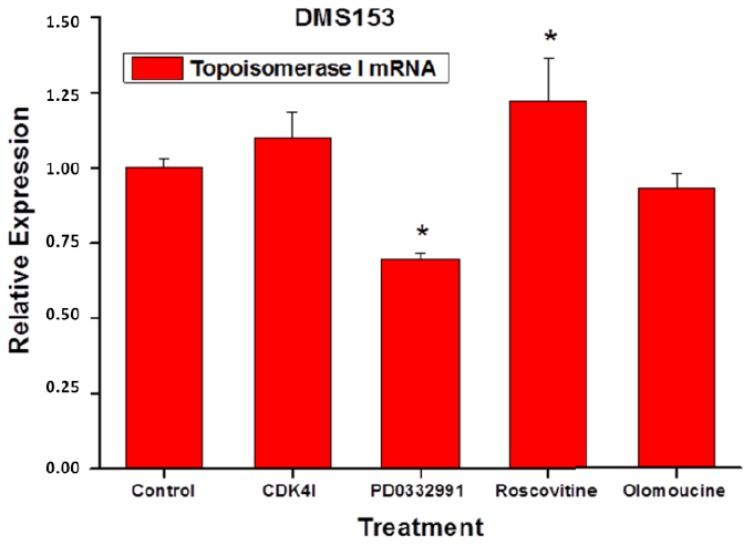
Effects of the CDK inhibitors on topoisomerase I (TOP1) mRNA expression was assessed by qPCR in DMS153 cells which were treated with the inhibitors for four days (mean ± SD, *n* = 2).

To assess possible effects of the CDK inhibitors on topoisomerase I (TOP1) mRNA expression DMS153 cells were treated with the inhibitors for four days and therafter gene expression quantified using qPCR (Figure 7). 2.5 µM CDKI and 25 µM olomoucine had no significant effects on TOP1 expression, whereas 2.5 µM PD0332991 reduced and 10 µM roscovitine increased expression of this gene, respectively.

### 2.7. Discussion

A broad range of CPT analogs has been developed to improve solubility and toxicity profile of the parent drugs CPT and HOCPT. However, today only TPT, mainly for second-line therapy of SCLC and ovarian cancer, and irinotecan for colon cancer are approved for treatment of cancer patients. Due to comparably low response rates and dose-limiting toxicities there is room left for improved chemotherapy in combination with TPT. One option would be the enhancement of cytotoxicity of the drug with modulators of low toxicity, targeting molecular events participating in the anticancer mechanisms of the drug. In the present study we have examined the cytotoxicity of a panel of CPTs with small molecule alterations against three SCLC cell lines of different degrees of chemoresistance. Of the clinically applicable CPTs, SN38, 9AC and rubitecan exhibited high cytotoxicity *in vitro*, followed by TPT and CPT109, the latter showing impared activity by adjacent modifications at positions 9 and 10. CPT-induced cell cycle perturbations were relatively small in chemoresistant NCI-H417 cells and more pronounced in less chemoresistant DMS153 cells. Differences in phase-specific accumulation in response to the CPT analogs point to slightly aberrant modes of action, especially for TPT, 9AC and CPT109. This was corroborated by assessment of the number of subG1 apoptotic cells, which showed large differences, either associated with the mode of action, efficacy or cell kill kinetics.

In the present work we employed several CDK inhibitors at concentrations that exhibited low toxicity in order to investigate the putative modulation of CPT cytotoxicity against the three SCLC cell lines. CDK4I is a cell-permeable, unsymmetrical indolocarbazole compound that displays anti-proliferative properties through reversible and ATP-competitive inhibition of CDK4. This inhibitor blocks tumor cells growth by impairing retinoblastoma susceptibility protein (Rb) phosphorylation and inducing G1 cell cycle arrest. Olomoucine is a purine derivative which inhibits CDK1, 2, 5, 7 and induces G1 arrest, and roscovitine/seliciclib has the same specificity as olomoucine, but shows 10-fold improved efficacy in stabilization of p53 as well as induction of cell cycle arrest and apoptosis [15]. The CDK inhibitors used in this study reduced G2M cells in NCI-H417 and caused accumulation mainly in G1/0 phase, whereas in DMS153 cells they showed different effects, with minor G1/0 arrest by CDK4I and roscovitine and G2M accumulation by PD0332991 and olomoucine. For the clinically employed CPTs, a concurrent and marked chemosensitization was observed in the order of 9AC, SN38, rubitecan and TPT.

Our results demonstrating the chemosensitizing effects of CDK inhibitors on the CPT-sensitivity of these two chemoresistant SCLC cell lines corroborate the findings obtained in NSCLC upon manipulation of the CDKN2A inhibitor of CDKs [16]. The mechanisms underlying the synergism of CPTs and the CDK inhibitors are not clear. During proliferation, progression through the cell cycle is first regulated at G1 by cyclin D1 and CDK4/6, which are expressed continuously throughout the early G1 phase before cells pass through the restriction point and enter S phase. If DNA repair is compromised, cells commit to exit G1 phase and proceed to undergo apoptotic cell death [17]. The tumor suppressor p16ink4 specifically binds and inhibits CDK4/6, preventing activation of the suppressor protein RB by phosphorylation. This tumor suppressor circuit is eliminated in SCLC [18,19]. Furthermore, NCI-H417 and DMS153 feature mutated p53 [20,21]. Since the DNA-damage checkpoint depends on p53 activation, the status of p53 might critically influence the response to CPTs. For the p53-mutated colon cancer cell line HT29 gain of sensitivity to irinotecan was increased by either restoration of wild-type p53 function or by sequential treatment with the roscovitine CDK inhibitor [22]. In summary, since the p16ink4/CDK4/6/p53 pathway is inactive in the SCLC cell lines it is not clear whether the chemosenitizing effects of the different CDK inhibitors is related to their impact on CDKs. The chemosensitizing effect of CDKs in combination with CPTs seems not to be related directly to the expression of the TOP1 gene, except in case of the CDK inhibitor PD0332991, which yields a significant reduction in TOP1 expression, possibly correlated with a lower synergistic effect with CPTs. A minor increase of TOP1 expression is significant for roscovitine treatment solely. A chemosensitizing effect of the CDK inhibitors in combination with CPTs is not likely to be mediated via P-glycoprotein/MDR-mediated chemoresistance due to the different structures of the inhibitors as well as the different CPTs and the inability of the same inhibitors to increase cytotoxicity of doxorubicin, a classical P-glycoprotein substrate (data not shown).

## 3. Experimental

### 3.1. Reagents and Cell Lines

Stock solutions of all compounds were prepared in DMSO. All chemicals were purchased from Sigma-Aldrich (St. Louis, MO, USA), except indicated otherwise. The PD0332991 inhibitor was obtained from Merck (Darmstadt, Germany). qPCR materials were purchased from Applied Biosystems (Foster City, CA, USA) and Quiagen (Hilden, Germany). NCI-H417 cells were obtained from ATCC (Rockville, MD, USA) and DMS153 cells from ECACC (Porton Down, Salisbury, UK). The SCLC26A line was established from a pleural effusion of an untreated patient with SCLC at our institution. Cells were grown in RPMI-1640 bicarbonate medium (Seromed, Berlin, Germany) supplemented with 10% fetal bovine serum (Seromed), 4 mM glutamine and antibiotics (10× stock formulated to contain ~5000 units penicillin, 5 mg streptomycin and 10 mg neomycin/mL) under tissue culture conditions (37 °C, 5% CO_2_, 95% humidity) and checked for mycoplasma contamination (Mycoplasma PCR ELISA, Roche Diagnostics, Vienna, Austria).

### 3.2. Chemosensitivity Assay

1 × 10^4^ cells in 100 μL medium per well were distributed in 96-well microtiter plates (Greiner, Kremsmuenster, Austria) and the test compound added in another 100 μL. Drugs and solute controls were serially diluted in twofold steps in triplicate. The microtiter plates were incubated under tissue culture conditions for four days and cell viability was measured using a modified MTT (3-(4,5- dimethylthiazol-2-yl)-2, 5-diphenyltetrazolium bromide) assay (EZ4U, Biomedica, Vienna, Austria). Optical density was measured using a microplate reader at 450 nm with an empty well as reference. Values obtained from control wells containing cells and media alone were set to 100% proliferation. For tests of synergy, compounds were diluted individually and in combination, using the same initial concentrations.

### 3.3. Measurement of Cell Cycle Distribution

1 × 10^6^ cells per well were incubated with the respective compound in six-well plates for three days. Harvested cells were washed with PBS and fixed with 70% ethanol at −20 °C for 30 min, washed again, transferred into staining solution (20 µg/mL propidium iodide (PI), 5 µg/mL ribonuclease A, 0.05% Nonidet P40 in PBS) and incubated at room temperature overnight. Washed cells were analyzed by acquisition of 1 × 10^4^ cells by flow cytometry (Cytomics FC500, Beckman Coulter, Krefeld, Germany) at excitation and emission wavelengths of 488 and 675 nm, respectively. The proportion of subG1 (apoptotic) cells was obtained from the logarithmic PI histograms, and percentages of cells in cell cycle phases G1/0 (resting), S (DNA synthesis) and G2M (mitotic) were calculated from linear PI histograms using MultiCycle AV software (Phoenix Flow Systems, San Diego, CA, USA). Experiments were done in duplicate.

### 3.4. RT-PCR of Topoisomerase I in DMS153 Cells

Total RNA was extracted from CDK inhibitor-treated cell cultures and cell lines using TRI Reagent^®^ (Applied Biosystesms) according to the manufacturer’s instructions. Two μg of total RNA was reverse transcribed to cDNA with the high capacity cDNA reverse transcription kit (Applied Biosystems), using random hexamer primers as recommended by the manufacturer. The target gene amplification mixture contained 5 μL 2× TaqMan^®^ Gene Expression PCR Master Mix, 0.5 μL of the appropriate Gene Expression Assay, 10 ng template cDNA diluted in 2.5 μL nuclease free water and 2 μL nuclease free water. Thermal cycling conditions were as follows: 2 min at 50 °C, 10 min at 95 °C, 40 cycles of 15 s at 95 °C and 1 min at 60 °C. Fluorescence generation from TaqMan^®^ probe cleavage was measured with the StepOnePlus system (Applied Biosystems). Real-time RT-PCR for topoisomerase I was performed with the Hs00243257_m1 TaqMan^®^ Gene Expression Assays (Applied Biosystems).

### 3.5. Statistics

Statistical analysis was performed using two-tailed Student’s t-test for normally distributed samples (* *p* < 0.05 was regarded as statistically significant). 

## 4. Conclusions

In conclusion, despite the mechanisms of interaction of the CDK inhibitors with CPT analogs, as employed in the present study, is not known, this synergism in drug-resistant SCLC lines is expected to improve the efficacy of clinically applicable CPTs and possibly lead to improved anticancer therapy in SCLC.
